# Cardioprotection by early metoprolol- attenuation of ischemic vs. reperfusion injury?

**DOI:** 10.1007/s00395-020-0814-2

**Published:** 2020-08-03

**Authors:** Petra Kleinbongard

**Affiliations:** Institute for Pathophysiology, West German Heart and Vascular Center, University of Essen Medical School, Hufelandstr. 55, 45122 Essen, Germany

There is still a need for adjunct cardioprotection on top of timely reperfusion since mortality and morbidity, notably from heart failure, in patients with acute myocardial infarction remain high [11, 12]. There are plenty preclinical studies reporting mechanical and/or pharmacological strategies to reduce myocardial ischemia/reperfusion injury [6, 17]. However, the translation from preclinical and clinical proof-of-concept studies into better clinical outcome for patients with acute myocardial infarction has been disappointing so far [9, 13]. Among the pharmacological strategies, the beta-blocker metoprolol when given after 15 min of a total of 90 min coronary occlusion reduced infarct size and improved left ventricular functional recovery in pigs [15]. Also, in a proof-of-concept study in patients with acute ST segment elevation myocardial infarction (The Effect of Metoprolol in Cardioprotection During an Acute Myocardial Infarction (METOCARD-CNIC) trial) metoprolol when given as early as possible i.e. in the ambulance -, reduced infarct size [14]. However, the subsequent phase III trial (the Early Intravenous Beta-Blockers in Patients With ST-Segment Elevation Myocardial Infarction Before Primary Percutaneous Coronary Intervention (EARLY-BAMI) trial) did not confirm the prior study and was neutral for infarct size and left ventricular function [20]. In retrospect, it turned out that the dose of metoprolol was lower and the start of treatment later in EARLY-BAMI than METOCARD-CNIC [5]. The importance of early treatment was now systematically addressed in an experimental study by Ibanez and collaborators [19].

In a closed-chest anesthetized pig model the distal left anterior descending coronary artery was occluded with an intracoronary balloon for varying durations from 20 to 60 min with subsequent reperfusion. Intravenous metoprolol (0.75 mg/kg) was given at 20 min coronary occlusion. Effects on ventricular fibrillation, infarct size, coronary microvascular obstruction and left ventricular function (by magnetic resonance imaging) were assessed at days 7 and 45. Metoprolol reduced ventricular fibrillation and improved survival. Metoprolol had no effect on infarct size with coronary occlusion of short duration (20 and 25 min). On the other extreme, with 60 min coronary occlusion, infarct size occupied almost the entire area at risk (determined by contrast multidetector computed tomography), and metoprolol also had no effect. However, in the time window from 30 to 50 min coronary occlusion, metoprolol significantly reduced infarct size. This infarct size reduction was equivalent in magnitude to a delay in infarct progression of about 15 min. Metoprolol reduced coronary microvascular obstruction, which was surprisingly small, and this effect was only significant with 45 min coronary occlusion. Also, metoprolol improved LV ejection fraction at 45 days reperfusion. The entire study was done on a background of amiodarone during ischemia, clopidogrel at reperfusion and oral metoprolol during reperfusion, reflecting a clinically relevant scenario. The study used state-of-the-art methodology [1, 18] and addresses both, a clinically and a conceptually important issue.

From a pragmatic point of view, the clinical message is very clear: metoprolol must be given as early as possible to reduce infarct size, and the present experimental study therefore somewhat reconciles the METOCARD-CNIC and EARLY-BAMI trials.

The conceptual issue, however is more difficult. At first glance, the authors` data support the notion that metoprolol delays ischemic damage rather than reduces reperfusion injury [21]. Indeed, with the unequivocal recognition of irreversible reperfusion injury, the existence of which had been debated for a long time, focus has been largely on the reduction of reperfusion injury, and ischemic injury tended to be neglected [7]. However, there currently exists no method which permits the reliable quantification of ischemic injury without reperfusion [1]. A recent study on remote ischemic perconditioning in pigs reported attenuated ST-segment elevation during ongoing coronary occlusion, thus supporting the idea of a reduction in ischemic rather than reperfusion injury, but a reduction of infarct size could also be ascertained only after subsequent reperfusion [16].

Therefore, there is an alternative interpretation of the present data. The extent of reperfusion injury depends on the duration of the preceding ischemia: with ischemia of short duration, there is also little reperfusion injury. With ischemia of long duration, there is little myocardium left which can be salvaged by reperfusion but also little myocardium that can suffer from reperfusion injury. Thus, reperfusion injury has a maximum at ischemia of intermediate duration [7]. The fact that metoprolol in the present study provided no protection at short duration ischemia (20 and 25 min) and at long duration ischemia (60 min) but did so at intermediate duration of ischemia would fit well to the time course of reperfusion injury which metoprolol could have attenuated. In each case, also metoprolol needed to be on board for more than 10 min to achieve a sufficient tissue concentration.

By definition, coronary microvascular obstruction becomes only manifest with reperfusion [8, 10]. Again, however, it is unclear whether the respective vascular damage is done during ischemia or during reperfusion. Metoprolol reduced coronary microvascular obstruction. In the absence of heart rate reduction, one would have expected a further reduction of coronary flow by beta-blockade [3]. Apparently, however, the attenuation of neutrophil plugging by metoprolol outweighed the vasoconstrictor effects of unmasked alpha-adrenoceptor activation [2].

Currently, it is unclear whether the observed cardioprotective properties of metoprolol can be extrapolated to other beta-blockers. However, the concept of early beta-blockade to salvage ischemic-reperfused myocardium from infarction has been advocated already for timolol more than three decades ago [4].
